# Antibacterial Activity Composition of the Fermentation Broth of *Streptomyces djakartensis* NW35

**DOI:** 10.3390/molecules18032763

**Published:** 2013-03-01

**Authors:** Wenjuan Zhang, Shaopeng Wei, Jiwen Zhang, Wenjun Wu

**Affiliations:** 1 College of Sciences, Northwest Agriculture and Forestry University, Yangling 712100, China; 2 State Key Laboratory of Crop Stress Biology in Arid Areas, Institute of Pesticide Science, Northwest Agriculture and Forestry University, Yangling 712100, China

**Keywords:** *Streptomyces djakartensis* NW35, (*E*)-2-methoxy-1,4 naphthoquinone-1-oxime, antibacterial activity

## Abstract

The new compound **Z-4-2** was isolated from the fermentation broth of *Streptomyces djakartensis* NW35, together with the known compound *N*-acetyltryptamine (**Z-9-2**) by bioassay-guided fractionation. Its chemical structure was elucidated as (*E*)-2-methoxy-1,4 naphthoquinone-1-oxime (**Z-4-2**) mainly by NMR analyses and MS spectral data. Their antibacterial activities against bacteria were evaluated by the filter paper method. The results of indicated that these compounds possess significant antibacterial activities.

## 1. Introduction

Soil actinomycetes are still important sources of novel antibiotics. The vast majority (70%) of the known antibiotics were first isolated from actinomycetes [1,2]. In the past decades, although many species which produced biologically active metabolites have been obtained from soil samples, the chance of isolating a new actinomycete strain from a common terrestrial habitant has reduced markedly [3,4]. To meet the increased demands for the discovery of new bioactive compounds, researchers have to look for novel microorganisms in unusual environments [5]. Chemically polluted soil is one such sort of unusual environment. In fact, chemical pollutants, especially some pesticides, could be mutagens, so mutant strains might be produced by induced mutations. Some of the mutant strains might give rise to increased productivity of bioactive metabolites, or even produce new bioactive compounds. Based on this thinking, the NW35 strain of *Streptomyces djakartensis* which was isolated from a pesticide-polluted soil sample has been investigated. In this paper, we report the antibacterial composition of the fermentation broth of *S. djakartensis* NW35.

## 2. Results and Discussion

### 2.1. Chemistry

**Figure 1:**
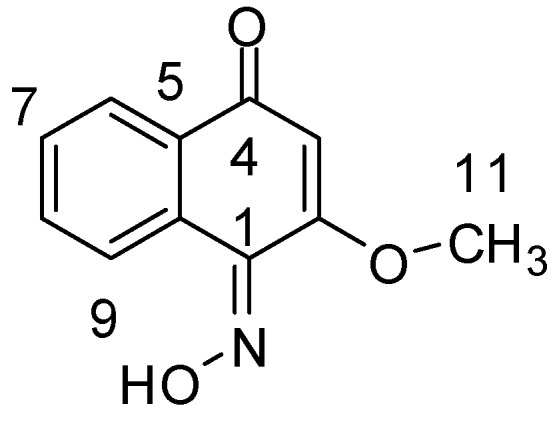
Structure of compound **Z-4-2**.

Compound **Z-4-2** (Figure 1) was obtained in the form of a yellow powder with m.p. 187–189 °C. Its molecular formula was determined as C11H9NO3 based on the HR-ESI-MS result with a *quasi* molecular ion peak of [M+H]^+^ at *m/z* 204.0657 (calcd. 204.0661). The IR spectrum of **Z-4-2** showed absorptions of hydroxyl (3546 cm^−1^) and conjugated carbonyl (1640 cm^−1^) moieties. The ^1^H-NMR spectrum of **Z-4-2** revealed the presence of four aromatic protons at *δ*_H_ 7.20 (1H, d, *J* = 8.5 Hz), 7.59 (1H, t, *J* = 8.5 Hz), 7.24 (1H, t, *J* = 8.5 Hz), 8.10 (1H, d, *J* = 8.5 Hz) and one hydroxyl proton at *δ*_H_ 9.75 (1H,brs), one olefinic proton at *δ*_H_ 6.16 (1H, s), one methoxy proton at *δ*_H_ 3.89 (3H, s). The ^13^C-NMR spectrum of **Z-4-2** exhibited 11 carbon signals, which were resolved through a DEPT experiment into one methyl, five methine, and five quaternary carbons (Table 1).

**Table 1:** ^1^H-NMR (500 MHz, CDCl_3_) and ^13^C-NMR (125 MHz, CDCl_3_) data for **Z-4-2**.

Position	^1^H-NMR	^13^C-NMR	HMBC
1		164.8	
2		160.8	
3	6.16 (1H, s)	105.2	C-1
4		182.6	
5		124.9	
6	8.10 (1H, d, *J* = 8.5 Hz)	131.4	C-4
7	7.24 (1H, t, *J* = 8.5 Hz)	124.2	
8	7.59 (1H, t, *J* = 8.5 Hz)	134.9	
9	7.20 (1H, d, *J* = 8.5 Hz,)	119.9	
10		136.9	
11	3.89 (3H, s)	56.6	C-2

**Figure 2:**
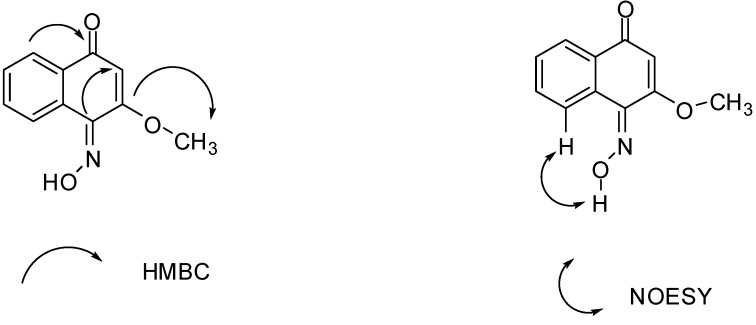
Key HMBC and NOESYof compound **Z-4-2**.

HMBC correlations from the conjugated carbonyl *δ*_C_ 182.57 to the aromatic proton at *δ*_H_ 8.10 indicated the presence on an enone connected to an aromatic carbon. Another key correlation signal from OMe to enone quaternary carbon at *δ*_C_ 160.78 indicated that is an β-OMe enone. Finally, the imine quaternary carbon signal *δ*_C_ 164.75 correlates with the olefinic proton *δ*_H_ 6.16 of the β-OMe enone, indicating that the imine quaternary carbon was connected with the β-OMe enone at the β position (Figure 2). Based on the above analysis, compound **Z-4-2** was assigned as 2-methoxy-1,4 naphthoquinone 1-oxime. A NOESY correlation signal can be observed between the hydroxyl proton *δ*_H_ 9.75 connected with the aromatic carbon at *δ*_H_ 7.20 indicated that the stereochemistry of the 4-hydroxyimino group that compound **Z-4-2** was (*E*) (Figure 2). (*E*)-2-Methoxy-1,4 naphthoquinone1-oxime (**Z-4-2**) is a new compound.

**Figure 3:**
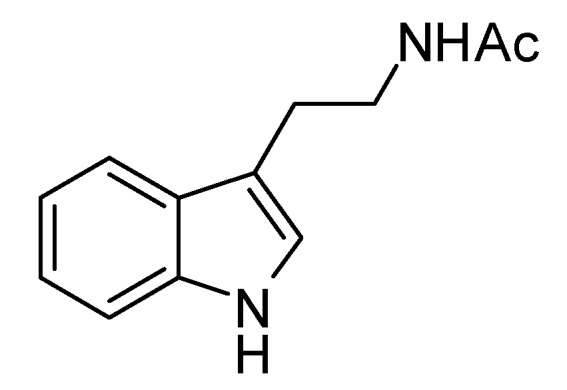
Structure of compound **Z-9-2**.

Compound **Z-9-2** (Figure 3) was obtained in the form of a yellow-brown oil. Its ESI-MS had a [M+H]^+^ molecular ion peak at *m/z* 203. The ^1^H-NMR spectrum of **Z-9-2** revealed the presence of four aromatic protons at *δ*_H_ 7.54 (1H, d, *J* = 9.0 Hz), 7.06 (1H, t, *J* = 8.0 Hz), 6.99 (1H, t, *J* = 8.0 Hz), 7.31 (1H, d, *J* = 7.5 Hz) and one indole proton at *δ*_H_ 7.05 (1H,s), one methyl proton at *δ*_H_ 1.89 (3H,s), two methylene proton at *δ*_H_ 2.92 (2H, t, *J* = 7.0 Hz), 3.45 (2H, t, *J* = 7.0 Hz). The ^13^C-NMR spectrum of **Z-9-2** exhibited 12 carbon signals, which were resolved through a DEPT experiment into one methyl *δ*_C_ 22.6, two methylenes *δ*_C_ 41.6, 22.6, five methines *δ*_C_ 112.2, 119.2, 119.6, 122.3, 123.3, and four quaternary carbons *δ*_C_ 173.2, 138.2, 128.8, 113.3. The above spectral data agreed with the literature values of *N*-acetyltryptamine [6] (Figure 3).

### 2.2. Bioactivity

The antibacterial activities of these two compounds against several strains of bacteria were evaluated by the filter paper method. The results indicated that compound **Z-4-2** and **Z-9-2** showed significant antibacterial activities (Table 2).

**Table 2:** Antibacterial activities of compounds **Z-4-2** and **Z-9-2** *in vitro*.

Test bacteria	Diameter of inhibition zone (mm) in 10 μL/disk (Mean ± S.D.)		
	Z-4-2	Z-9-2	Ampicillin
*Bacillus cereus*	11 ± 0.3 (+++)	12 ± 0.2 (+++)	14 ± 0.4 (+++)
*Bacillus subtilis*	13 ± 0.2 (+++)	15 ± 0.4 (+++)	16 ± 0.3 (+++)
*Staphylococcus aureus*	8 ± 0.3 (+++)	9 ± 0.5 (+++)	15 ± 0.1 (+++)
*Escherichia coil*	9 ± 0.8 (+)	10 ± 0.3 (+)	13 ± 0.2 (+)
*Pseudomonas aeruginosa*	-	-	7 ± 0.1 (+)
*Pseuomonas syringae pv. Actinidiae*	15 ± 0.1 (++)	17 ± 0.3 (++)	11 ± 0.6 (+++)
*Erwinia carotovora*	-	-	-
MRSA	13 ± 0.2 (++)	12 ± 0.2 (++)	17 ± 0.4 (+++)

Note: All values were means of three replicates, “+” means visible; “++” means clear; “+++” means transparent; “-” means no inhibitory ring or no inhibition activity.

## 3. Experimental

### 3.1. General

Melting points (uncorrected) were taken on a Fisher-Johns melting point apparatus. IR spectra were acquired using a Bruker TENSOR2 spectrophotometer. The NMR data were recorded on a Bruker Avance 500 instrument (500 MHz for ^1^H and 125 MHz for ^13^C) in CDCl_3_ with TMS as internal standard. HR-ESI-MS data were obtained by Bruker Daltonics APEX II 49e (ESI) mass spectrophotometer. Silica gel (200–300 mesh, Qingdao Haiyang Chemical Co. Ltd, Qingdao, China) was used for chromatographic separations.

### 3.2. Microorganism and Fermentation

*Streptomyces djakartensis* NW35 was isolated from a polluted soil sample collected in Qingdao, Shandong Province, China, and identified by its morphology, physiology, biochemistry, and 16S rDNA gene sequence. The voucher specimen of this streptomycete was deposited in the Institute of Pesticide Science, Northwest Agriculture and Forestry University, Yangling, China. *S. djakartensis* NW35 was cultivated at 28 °C in starch casein agar medium, which contained soluble starch (1%), K_2_HPO_4_ (0.2%), KNO_3_ (0.2%), NaCl (0.2%), Casein (0.03%), MgSO_4_ (0.005%), CaCO_3_ (0.002%), FeSO_4_ (0.001%) and agar (1.5%). Fermentation was performed in two stages: seed growth and antibiotics production. The spores of *S.* *djakartensis* NW35 grown on starch casein agar were used to inoculate a 250 mL flask containing 60 mL of a sterile seed medium consisting of glucose (1.0%), millet steep liquor (1.0%), peptone (0.5%), (NH_4_)_2_SO_4_ (0.1%), NaCl (0.25%),and CaCO_3_ (0.05%); pH 7.2. The flask was shaken on a shaker at 180 rpm for 18 h at 28 °C. Six mL of the seed culture were transferred to 250 mL flasks containing 60 mL of a sterile production medium consisting of glucose (1.0%), millet steep liquor (1.0%), peptone (0.3%), (NH_4_)_2_SO_4_ (0.1%), NaCl (0.25%) and CaCO_3_ (0.1%); pH 7.2. Fermentation was carried out at 180 rpm for 4 days at 28 °C on a rotary shaker.

### 3.3. Extraction and Isolation

The culture of 90 L was filtered through cheesecloth to separate the medium and culture liquid at 25 °C, pH 7.0. The filtrate was absorbed onto HPD100 macroporous resin (Baoen Co., Ltd., Cangzhou, Hebei, China), and then eluted with H_2_O and MeOH in sequence. The MeOH fraction was evaporated in vacuum. The concentrate was subjected to column chromatography and eluted with EtOAc and MeOH in sequence. The antimicrobial fraction was concentrated under vacuum, and further purified on a Waters 600E HPLC apparatus (Waters Co., Ltd., Milford, MA, USA) equipped with a Hypersil ODS-BP (20 × 250 mm, 10 μm) reverse phase column, using methanol-water as the mobile phase, flow rate of 3.0 mL/min, monitored by UV detector at 230 nm to afford two compounds **Z-4-2** (20 mg) and **Z-9-2** (10 mg).

### 3.4. Antibacterial Activity

The standard bacterial strains *Bacillus cereus* (1.1846), *Bacillus subtilis* (1.88), *Staphylococcus aureus* (1.89), *Escherichia coil* (1.1574), and *Pseudomonas aeruginosa* (1.2031) were obtained from China General Microbiological Culture Collection Center. *Pseuomonas syringae pv. Actinidiae*, *Erwinia carotovora* were obtained from College of Plant Protection, Northwest A & F University. A clinical isolate of MRSA was obtained from Nanjing Medical University. Ampicillin (Sigma, Shanghai, China) was used as positive control. The antibacterial activities of compounds against eight strains of bacteria were evaluated by the filter paper method [7], Mueller-Hinton (Hangzhou Microbial Reagent Co. Ltd., Hangzhou, China) agar was used as an assay medium. The medium at 45 °C was mixed with the pathogen bacterial suspension containing approximately 10^8^ cfu·mL^−1^. Next, the mixture was poured on 9 cm Petri dishes. The tested compounds were dissolved in acetone at the concentration of 1,000 ppm, The filter paper (5 mm in diameter) were impregnated with 10 μL/disc of each compound, then were absolutely dried and placed on the inoculated agar. The inoculated plates were incubated at 37 °C for 24 h. Antibacterial activity was evaluated by measuring the zone of inhibition against the test organism. Experiments were run in triplicate. The results indicated that **Z-4-2** and **Z-9-2** could effectively inhibit Gram-positive bacteria, such as *Bacillus cereus*, *B. subtilis*, whereas they were inactive towards Gram-negative bacteria (Table 2).

## 4. Conclusions

Two compounds (*E*)-2-methoxy-1,4 naphthoquinone-1-oxime (**Z-4-2**) and *N*-acetyltryptamine (**Z-9-2**) were isolated from the extract of fermented broth of *Streptomyces djakartensis* NW35 by bioassay-guided fractionation. (*E*)-2-Methoxy-1,4 naphthoquinone-1-oxime (**Z-4-2**) is a new natural product. These two compounds both showed significant antibacterial activities against *Bacillus cereus*, *Bacillus subtilis*, *Staphylococcus aureus*, *Escherichia coil*, *Pseuomonas syringae pv. Actinidiae* and MRSA.
